# Tracing the origin and evolution of specialized biosynthetic pathways in marine organisms

**DOI:** 10.1128/mbio.03375-25

**Published:** 2026-01-27

**Authors:** Laure Martinelli, Nicolas Papon, Olivier P. Thomas, Vincent Courdavault

**Affiliations:** 1 Biomolécules et Biotechnologies Végétales, BBV, EA2106, Université de Tours 27092, Tours, France; 2 Univ Angers, Univ Brest, IRF, SFR ICAT 26995, Angers, France; 3 School of Biological and Chemical Sciences, Ryan Institute, University of Galway 219560, Galway, Ireland; Georgia Institute of Technology, Atlanta, Georgia, USA

**Keywords:** microalgae, biosynthesis, evolution, kainoids, toxins

## Abstract

Harmful algal blooms (HABs) pose public health and ecological risks in aquatic environments. HABs drive the bioaccumulation of a specific family of specialized metabolites known as “kainoids.” Kainoid derivatives, such as kainic acid (KA) and domoic acid (DA), are among the most toxic marine-derived metabolites produced by a limited number of algal species. While recent studies have provided insights into the molecular basis of KA and DA production in red algae and diatoms, knowledge of the biosynthesis of kainoids remains insufficient. In a new report published in *mBio*, Wood-Rocca et al. decode the DA biosynthetic route in the widespread Western Pacific benthic diatom *Nitzschia navis-varingica* (S. M. Wood-Rocca, N. Allsing, Y. Ashida, M. Mochizuki, et al., mBio 16:e02079-25, 2025, https://doi.org/10.1128/mbio.02079-25). We discuss how evolutionary genomics studies bridge the gap between fundamental biology and applied environmental and biotechnological research, enhancing our ability to understand, predict, and harness marine natural products.

## COMMENTARY

Plants, fungi, algae, and bacteria biosynthesize a myriad of specialized metabolites, reflecting millions of years of ecological pressures and evolutionary changes. Many of these natural compounds have become landmarks of modern medicine, as illustrated by aspirin, penicillin, and anticancer drugs such as paclitaxel and vinblastine (1). This large natural chemical space encompasses notorious toxins that threaten animals including humans, as exemplified by mycotoxins contaminating cereals and phycotoxins found in seafood (2). Contamination of marine seafood is increasingly associated with harmful algal blooms (HABs)—events defined as the short-term proliferation and dominance of toxic or harmful algae—which drive the bioaccumulation of a specific family of specialized metabolites known as “kainoids” (2). Over the past 50 years, the occurrence of these toxic blooms has had a significant negative economic impact and has posed an increasing risk to humans and animals. Kainoids, including kainic acid (KA) and its analogs such as domoic acid (DA), act as glutamate agonists in the central nervous systems of vertebrates and in other organs with high concentrations of glutamate receptors. Consumption of kainoid-contaminated sea products can cause amnesic shellfish poisoning, which manifests gastrointestinal and neurological symptoms (e.g.*,* memory impairment, seizures, and epilepsy) and can occasionally be fatal in mammals. These kainoid neurotoxins commonly feature a characteristic pyrrolidine scaffold derived from glutamate and an isoprenoid unit. Their production has mainly been observed in species of algae belonging to two distantly related taxa: the Rhodophyta (also known as “red algae”) and the Bacillariophyta (photosynthetic diatoms). While their occurrence and toxicity are well documented, the molecular mechanisms underlying their biosynthesis have begun to be unraveled only recently.

**Fig 1:**
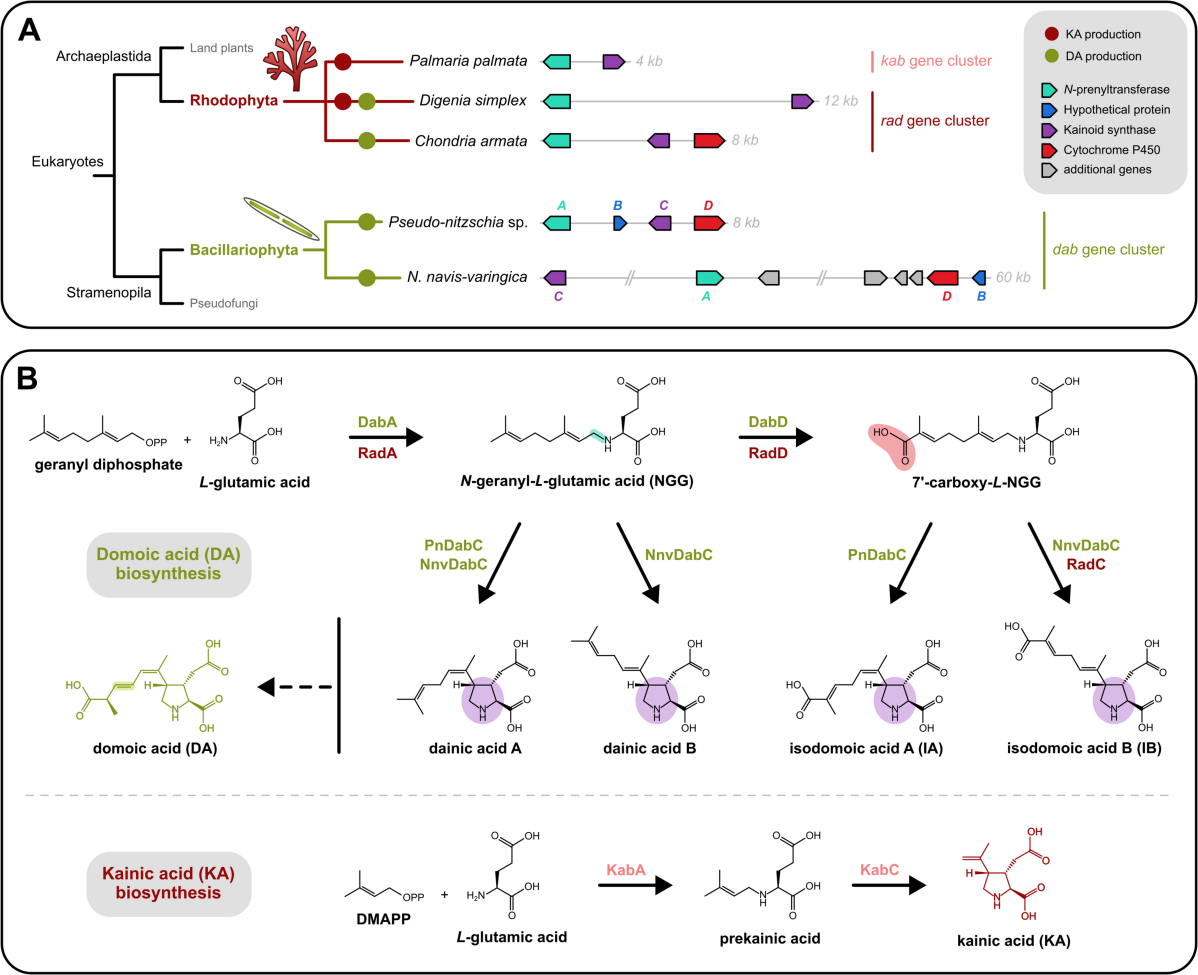
Genomic organization and enzymatic pathway of domoic acid (DA) and kainic acid (KA) biosynthesis across algal taxa. (**A**) Schematic overview of kainoid-producing species and a representative synteny map of their associated biosynthetic gene clusters (BGCs). Species shown include the KA-producing red algae *Digenea simplex* and *Palmaria palmata*, the DA-producing red alga *Chondria armata* (Rhodophyta), and DA-producing species from the genera *Pseudo-nitzschia* and *Nitzschia* (Bacillariophyta). Only the general structure and gene order of the BGCs are depicted, based on the architectures described in Wood-Rocca et al*.* (4). The *Pseudo-nitzschia* BGC corresponds to a consensus representation derived from *P. australis*, *P. cuspidata*, *P. multiseries*, *P. multistriata*, and *P. seriata*. For *Nitzschia navis*-varingica, the cluster is shown in truncated form for readability: the genomic distances between *dabC-dabA* and *dabA-dabD* are each approximately three times longer in the native locus. In this cluster, more coding regions have been identified and correspond to additional genes potentially involved in the regulation of DA biosynthesis. (**B**) Overview of the biosynthetic pathways and enzymes involved in DA and KA production. Biosynthesis begins with the condensation of l-glutamate (Glu) with an isoprenoid unit—either geranyl diphosphate (GPP) or dimethylallyl diphosphate (DMAPP)—catalyzed by an *N*-prenyltransferase (DabA, RadA, or KabA). This is followed by cyclization by a kainoid synthase (DabC, RadC, or KabC) to produce DA or KA. In DA-producing species, the monoterpene-derived alkyl chain (from GPP) enables an oxidation, before or potentially after the cyclization, by a cytochrome P450 enzyme (DabD and RadD), which installs the terminal carboxylic acid.

The first enzymes involved in kainoid biosynthesis were characterized in 2018 (3), in a species belonging to the *Pseudo-nitzschia* genus*,* a pennate diatom known for its association with HABs and for producing DA, a kainoid analog. In this work, Bradley Moore’s team discovered a biosynthetic gene cluster (BGC) comprising four genes involved in the production of DA. This domoic acid biosynthesis (*dab*) gene cluster encodes an *N*-prenyltransferase (*dabA*), a hypothetical protein found only in DA-producing diatoms (*dabB*), a kainoid synthase (*dabC*) involved in the cyclization step, and a cytochrome P450 (*dabD*). The genomic co-localization of these four *dab* genes was a key feature that enabled the pioneering identification of kainoid biosynthetic steps. Indeed, no enzyme family capable of generating the pyrrolidine core derived from glutamate and an isoprenoid unit had been hitherto identified (Fig. 1A). In addition, the cluster organization provided valuable insights into the emergence and evolution of kainoid biosynthesis. In this respect, analysis of the intergenic spaces suggested that a combination of horizontal gene transfer (HGT) events and enzyme neofunctionalization may account for the production of these neurotoxic compounds in phylogenetically distant marine species such as red algae.

Since then, homologous BGCs have been identified and compared across other *Pseudo-nitzschia* diatom species, as well as several red algae such as *Chondria armata*, *Digenea simplex,* and *Palmaria palmata* (5–7). Overall, the *dab* gene cluster (also referred to as the *rad* gene cluster, for “red algae DA biosynthesis” gene cluster and the *kab* gene cluster in species producing only KA) (Fig. 1A) is relatively conserved: the key genes required for DA or KA biosynthesis are present in both diatoms and red algae and consistently appear in the same order. However, *dabB* appears to be specific to diatoms, and *dabD* is absent from the non-DA-producing alga *P. palmata* (6, 7). Furthermore, analysis of the different clusters reinforces the hypothesis that BGC acquisition involved HGT and even suggests a common integration site (7). More recently, and beyond their fundamental evolutionary significance, the conserved genomic architecture of the *dab* gene cluster has been used as a molecular marker for bloom monitoring and the development of HAB-mitigation strategies (8). However, similar analyses are needed for other harmful algal species to enable effective and holistic HAB prevention.

In a new report published in *mBio*, Wood-Rocca et al. resolved the *dab* gene cluster organization in the benthic diatom *Nitzschia navis-varingica* (4). Species of the genus *Nitzschia* are among the most abundant species of Bacillariophyta involved in HABs, and, along with *N. bizertensis*, *N. navis-varingica* is one of the two species from this genus known to produce DA. The authors first sequenced three regionally distinct strains of the diatom using long-read technology (PacBio IsoSeq and PacBio HiFi) to obtain both genomic and transcriptomic data. By exploiting previous genomic resources from both Rhodophyta and Bacillariophyta toxin-producing algae, the authors identified the homologous *dab* gene cluster containing the four expected genes (*dabA-D*) in *N. navis-varingica* (Fig. 1A). Further functional characterizations of DabA and DabD, coupled with protein structure analyses, corroborated previous results (9). For instance, DabA from *N. navis-varingica* (NnvDabA) catalyzes the initial step, condensing l-glutamate and geranyl diphosphate to form *N*-geranyl-l-glutamic acid and NnvDabC is the kainoid synthase responsible for the cyclization into the pyrrolidine core (Fig. 1B). Unexpectedly, the newly characterized kainoid synthase, NnvDabC, leads to isodomoic acid B, as observed in red algae (RadC; Fig. 1B) rather than isodomoic acid A, as produced by a diatom kainoid synthase (PnDabC; Fig. 1B). Phylogenetic analyses combined with protein modeling indicate that the NnvDabC protein is more closely related to its ortholog found in the red alga *C. armata* (RadC) than to the DabC proteins present in *Pseudo-nitzschia* species. Their analyses also suggest that the presence of different intermediates within the biosynthetic pathway, such as dainic acids, may contribute to the kainoid biochemical diversity.

Nevertheless, the most notable finding reported by Wood-Rocca et al. (4) concerns the organization of the complete BGC. In comparison with the *dab* and *rad* gene clusters of other diatoms and red algae, the cluster identified in *N. navis-varingica* differs in size, organization, and composition. First, the four genes are present—but not in the same order (Fig. 1B). Then, the BGC spans approximately 60 kb, compared to the 8 kb-BGC observed in DA-producing diatoms and the 6 kb-BGC in the DA-producing red alga. This latter discovery explains why the previous study on *N. navis-varingica* conducted by Cui et al. (9) could not resolve the gene cluster using a short-read sequencing approach. Notably, this unique cluster contains additional single-copy coding regions, potentially involved in its regulation. These regions were likely integrated during expansion and rearrangement events, which also increased the overall amount of non-coding DNA containing repetitive elements. The expansion of the kainoid BGC in *N. navis-varingica* mirrors the species’ overall genome expansion, which is 15–22 times larger than that of other pennate diatoms and consists of roughly 70% repetitive DNA.

Overall, the results from Wood-Rocca et al. both expand and challenge the current knowledge of kainoid phycotoxins biosynthesis. While the *dab* gene cluster previously identified in DA-producing diatoms and red algae was also found in *N. navis-varingica*, this report highlights several unexpected evolutionary features of the kainoid biosynthetic pathway in diatoms. The presence of a *dabB* homolog in *N. navis-varingica* of unknown function stands out as a specific enzymatic innovation in the toxinogenic Bacillariophyta. Similarly, analysis of this new cluster further supports the involvement of HGT in the emergence of the *dab*/*rad*/*kab* gene cluster across distantly related marine taxa. Moreover, the functional divergence of NnvDabC not only drives structural diversification in kainoid chemistry but also points to an unprecedented evolutionary trajectory for kainoid synthases. Finally, the presence of additional and uncharacterized protein-encoding sequences in the *dab* cluster—and even more so in the larger version (60 kb) of *N. navis-varingica*—raises fundamental questions about their role and the adaptive significance of their co-localization with the *dabA-D* genes.

In conclusion, the report from Bradley Moore’s laboratory provides new perspectives into the origin, evolutionary path, and distribution of biosynthetic pathways of specialized metabolites across the tree of life. Beyond these evolutionary insights, this work could make a significant contribution to the development of blue biotechnology. Indeed, the ability to identify, trace, and use biosynthetic genes as molecular markers opens promising avenues for environmental diagnostics including early detection, precision monitoring, and targeted mitigation of harmful metabolites. Such innovations have the potential to substantially enhance food security and environmental resilience but also offer potential perspectives for the development of new bioactive compounds. Overall, this study demonstrates how evolutionary genomics can connect fundamental biology with applied environmental and biotechnological research, thereby strengthening our ability to understand, predict, and utilize marine natural products.

## References

[1] Newman DJ, Cragg GM. 2007. Natural products as sources of new drugs over the last 25 years. J Nat Prod 70:461–477. doi:10.1021/np068054v 17309302

[2] Guillotin S, Delcourt N. 2022. Marine neurotoxins’ effects on environmental and human health: an OMICS overview. Mar Drugs 20:18. doi:10.3390/md20010018 PMC8778346 35049872

[3] Brunson JK, McKinnie SMK, Chekan JR, McCrow JP, Miles ZD, Bertrand EM, Bielinski VA, Luhavaya H, Oborník M, Smith GJ, Hutchins DA, Allen AE, Moore BS. 2018. Biosynthesis of the neurotoxin domoic acid in a bloom-forming diatom. Science 361:1356–1358. doi:10.1126/science.aau0382 30262498 PMC6276376

[4] Wood-Rocca SM, Allsing N, Ashida Y, Mochizuki M, Moore ML, Füssy Z, Kotaki Y, Puilingi C, Maeno Y, Beattie AW, Allen AE, Yotsu-Yamashita M, Michael TP, Moore BS. 2025. Domoic acid biosynthesis and genome expansion in Nitzschia navis-varingica. mBio 16:e02079-25. doi:10.1128/mbio.02079-25 41165336 PMC12691652

[5] Chekan JR, McKinnie SMK, Moore ML, Poplawski SG, Michael TP, Moore BS. 2019. Scalable biosynthesis of the seaweed neurochemical, kainic acid. Angew Chem Int Ed 58:8454–8457. doi:10.1002/anie.201902910 PMC6574125 30995339

[6] Steele TS, Brunson JK, Maeno Y, Terada R, Allen AE, Yotsu-Yamashita M, Chekan JR, Moore BS. 2022. Domoic acid biosynthesis in the red alga Chondria armata suggests a complex evolutionary history for toxin production. Proc Natl Acad Sci USA 119:e2117407119. doi:10.1073/pnas.2117407119 35110408 PMC8833176

[7] He Z, Xu Q, Chen Y, Liu S, Song H, Wang H, Leaw CP, Chen N. 2024. Acquisition and evolution of the neurotoxin domoic acid biosynthesis gene cluster in Pseudo-nitzschia species. Commun Biol 7:1378. doi:10.1038/s42003-024-07068-7 39443678 PMC11499653

[8] Brunson JK, Thukral M, Ryan JP, Anderson CR, Kolody BC, James CC, Chavez FP, Leaw CP, Rabines AJ, Venepally P, Fussy Z, Zheng H, Kudela RM, Smith GJ, Moore BS, Allen AE. 2024. Molecular forecasting of domoic acid during a pervasive toxic diatom bloom. Proc Natl Acad Sci USA 121:e2319177121. doi:10.1073/pnas.2319177121 39298472 PMC11459128

[9] Cui Z, Wang H, Lim PT, Tan SN, Kotaki Y, Leaw CP, Chen N. 2025. Genomic and phylogenetic analyses of the domoic acid biosynthesis genes in the benthic diatom Nitzschia navis-varingica (Bacillariophyceae). Harmful Algae 148:102905. doi:10.1016/j.hal.2025.102905 40835329

